# Regulating artificial-intelligence applications to achieve the sustainable development goals

**DOI:** 10.1007/s43621-021-00064-5

**Published:** 2021-11-29

**Authors:** Hoe-Han Goh, Ricardo Vinuesa

**Affiliations:** 1 Institute of Systems Biology, UniversitiKebangsaan Malaysia, 43600 Bangi, Selangor Malaysia; 2 FLOW, Engineering Mechanics, KTH Royal Institute of Technology, 10044 Stockholm, Sweden; 3 AI Sustainability Center, 114 34 Stockholm, Sweden

## Abstract

Artificial intelligence is producing a revolution with increasing impacts on the people, planet, and prosperity. This perspective illustrates some of the AI applications that can accelerate the achievement of the United Nations Sustainable Development Goals (SDGs) and highlights some of the considerations that could hinder the efforts towards them. In this context, we strongly support the development of an 18^th^SDG on digital technologies. This emphasizes the importance of establishing standard AI guidelines and regulations for the beneficial applications of AI. Such regulations should focus on concrete applications of AI, rather than generally on AI technology, to facilitate both AI development and enforceability of legal implications.

## Introduction

The advent of information-and-communications technologies (ICT), as well as the Internet of Things (IoT), has culminated in the fourth industrial revolution (4IR) and ushered in the big-data era of artificial intelligence (AI)-enabled precision and personalization via the convergence of physical, biological, and digital technologies. AI is game changing, by maximizing objective functions for the optimal outcome. This perspective aims to showcase some of the well-established applications of AI and highlights some of the important considerations or issues related to the implementation of AI towards an accelerated achievement of the Sustainable Development Goals (SDGs). Below we discuss some areas where AI can have a significant impact.

For instance, in agriculture, AI allows many labor-intensive processes to be automated [1], including autonomous systems in transport, logistics, and supply chains. Smart systems can be deployed for farm monitoring, management, husbandry, operation, and surveillance, as well as for implementing smart contracts with blockchain for automated transactions. These systems have the advantages of transparency, reliability, efficiency, and cost savings. Speed breeding of crops is made possible with smart greenhouses regulating the photoperiods via LED lighting to shorten the time to flowering and fruiting. Automated vertical farms integrated with aquaculture have been adapted for urban farming to reduce the carbon footprint of transporting fresh produce. Farming on the rooftop of office buildings and skyscrapers not only maximizes space usage but also adds greenery to the urban landscape against pollution while harvesting solar energy for food production and generating renewable electricity with solar panels. Soil-less plantation methods such as aquaponics or aeroponics are no longer restricted to vegetables but also tuberous crops such as potatoes. High-tech sustainable farming with wastewater treatment for zero waste is in line with the circular economy and can even prolong crop shelflife.

AI-enabled precision farming integrates climate sensors with automated irrigation and fertilizing systems that canfunction efficiently with minimal labor. The digital twin of greenhouses permits real-time monitoring and controls. Smart sweepers or harvesters are made possible with continuous machine learning (ML) based on virtual training with augmented reality (AR) for robots to recognize fruits of different ripening stages from hard-to-reach areas. For large-scale farming, IoT-enabled monitoring of soil and weather conditions permits the automation of farm husbandry. Diverse robotic and drone technologies are available for weeding, fertilizing, seeding, pruning, and harvesting in the field. Satellite and drone imaging allow field assessment to inform agrichemical applications and measure crop productivity for yield prediction. The Governmental Agri-Food Transformation Fund will encourage the adoption of precisionfarming practices worldwide. Active partnerships of colleges with local farms can be forged via transformative project-based internships. Beyond that, workforce-upskilling initiatives are timely to be deployed via technical and vocational education and training (TVET) and micro-credentials for all sectors and industries. AI can contribute to personalized learning [2]for quality education.

While developing technology to increase food production with efficient farming, green citizenry should also be cultivated through awareness campaigns to reduce consumption and food waste. AI can help to reduce waste with a more efficient supply chain [3]. Automated food delivery to predict daily orders based on consumer big data for ingredient logistics and food-waste minimization promote a more sustainable food system. In particular, implementing the circular economy in the food system is of extreme importance [4]. The impacts will be tremendous when applied to large fast-food chains around the world to optimize resources. Another transformative change is to shift from animal-based to plant-based proteins, which will significantly reduce the emissions of greenhouse gases (GHGs). To change dietary preference, a Chilean company, NotCo, uses the AI algorithm “Giuseppe” to reproduce animal taste and texture based on molecularflavor profiles from hundreds of thousands of plant ingredients to look, cook, blend, and taste, similar to animal produce [5]. Such a food-tech disruptor can not only save the environment but also ensure food security for the future.

Agriculture is a critical component of national security and world health because food shortages will lead to famine and political unrest. At Descartes Labs [6], physicists use the principles of physics and light with remote sensing to predict when disease, disaster, or war might strike based on historical satellite imagery. The similarity-search engine allows object recognition for monitoring land usage. This enables nationwide prediction of crop yield in US cornfields and Asian rice paddies with crop health inferred from satellite infrared bands. Weather forecastcan also better prepare farmers with planning to minimize losses from possible drought or flood based on water-availability models. Earth-observation data can also help to understand socioeconomic and environmental trends to predict how today’s decisions will impact tomorrow’s society. It is possible to infer poverty and food-production situations in the Middle East and North Africa for pre-emptive food aids in times of crop failure to prevent famines months before they happen, especially in troubled areas. In case of a crisis, every nation needs a future-ready food hub for global coordination to assess and regulate food security and safety to ensure reliable sources of food supplies and accountability within the intertwined global foodsupply chain.

AI is also useful for environmental conservation to save wildlife, biodiversity and the world, with the goal of haltingthe sixth mass extinction. For example, Intel’s TrailGuard [7], which is equipped with a vision-processing unit (VPU), can detect a human from body shapes, facial geometry, gait, and movement from any angle and light condition via intelligentimage recognition. It serves as a hidden park ranger that works 24/7 tracking wildlife poachers with an automated alert system connected to the control center to prevent poaching before it happens. The current predictive models exhibit unprecedented precision through continuous data training. The models are everimproving without apriori settings for unsupervised analysis. AI can identify patterns for improving disaster or calamity prediction, a feature that can be used toprevent catastrophes. ShakeAlert® applies ML to discern noise from signals of earthquakes via hundreds of vibrational sensors scattered around large cities such as San Francisco and Seattle [8].

## Considerations and Issues of AI applications

**Fig. 1:**
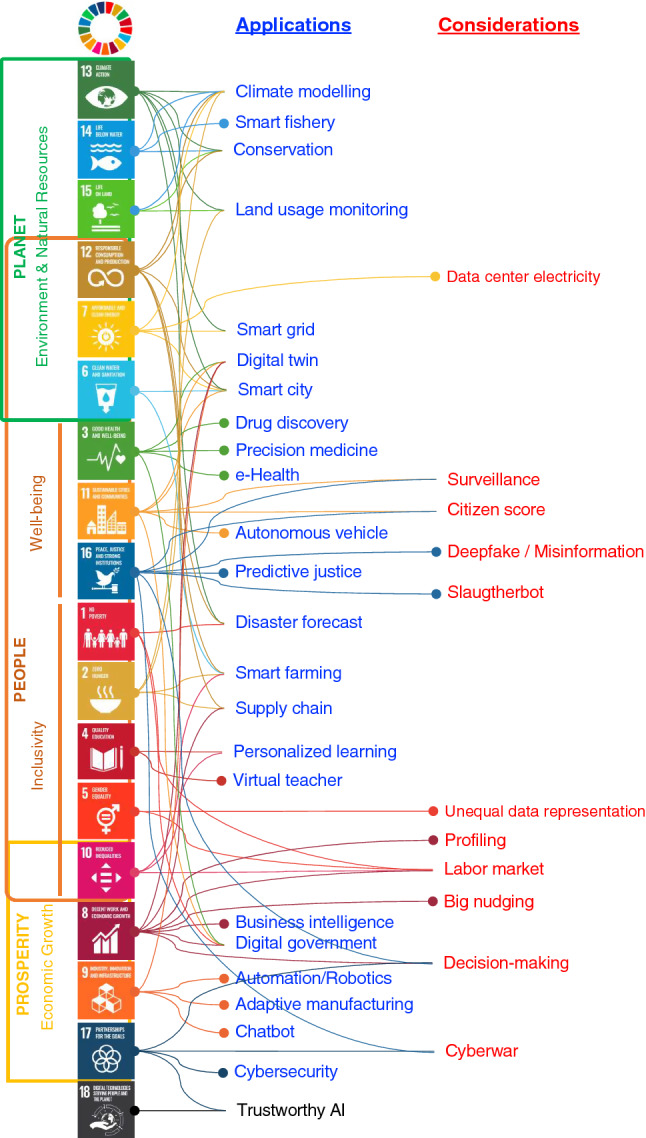
Applications and considerations of AI in relation to the 17 Sustainable Development Goals. The 18th SDG on Digital Technologies Serving People and the Planet wasproposed by the Montreal Statement on Sustainability in the Digital Age [12]. to ensure that the digital age supports people, the planet, prosperity, peace, and partnerships. Note that the arrangement and grouping of SDGs into planet, people, and prosperity have not been reviewed by the United Nations and do not reflect its views. The applications and considerations are not an exhaustive list to reflect digital interdependence. Some connections between the applications/considerations and SDGs are not shown for simplicity, as represented by the rounded ends

Even though AI technology can be an accelerator for achieving the United Nations Sustainable Development Goals (UN SDGs), it is essential to realize that AI is not a universal solution. AI solutions are often obtained through an evolutionary process instead of a turnkey method. Transformative solutions are much rarer than incremental solutions. Passionate, purpose-driven leaders are essential to champion sustainability as the main goal in organizations, society, local governments, and countries. In this respect, leaders and industry players from different sectors play important roles in the future of AI. Outlooks and perspectives on AI involve active discussions from all stakeholders on the issues, considerations, and implications of different AI applications (Fig. 1). AI could play both enabler and inhibitor roles in achieving the SDGs [9, 10]. Hence, holistic assessments will be needed for each AI application by considering the societal, economic, and environmental impacts of digital sustainability (digitainability) [11].

Some of the pressing issues and considerations include transparency, equality, automated decision making, and ethical standards of profiling [13]. These considerations have been extremely relevant in the context of digital contact tracing for pandemic management, as exemplified in the case of the COVID-19 pandemic [14]. Infrastructural vulnerability has shifted from the millennial digital divide to digital interdependence. It is inevitable for an accelerated trend of digitalization and AI applications to achieve the SDG indicators. Therefore, practical implications of AI-based technological deployment need to be considered beyond sectorial silos to reflect the cross-cutting and interconnectedness of SDGs (Fig. 1). For instance, the digital twin can be represented in multiple SDGs, while the exponentially increasing electricity demands of ICTs and data centers will need solutions from renewable-energy sectors [15]. This requires a systems approach to consider all interactions among the targets of all the SDGs [16, 17]. The current speed of technological advancements surpasses the individual adoption and governmental regulations of double-edged AI [10]. To reap the maximum potential from doing good with AI, such as smart fisheries [18], smart grids [19], smart cities [20], and e-health [21], we must impose controls on “big nudging”, citizen scores, and deepfake. All of this while balancing societal polarization with ethics. Gender inequality in data with male-dominated datasets and manpower should be clarified to avoid discrimination and bias in AI applications [22]. The same applies to datasets and deep-learning models biased against certain ethnicities [23].

Internet history leaves a trail of digital exhaust that is hoovered for behavioral prediction and microtargeting with the possibility of becoming a social contagion, as was the case with election manipulation by Cambridge Analytica via Facebook [24]. Such a scenario is a reminder to government policymakers and regulators to think twice about the aggressive lobbying of tech giants against regulations. Harmful misuses must be prevented via international dual-use aware policy and regulations such as that of the Biological Weapons Convention [25].

## Regulating AI

Countermeasures should be implemented against AI terrorism, such as cyber-attacks, slaughterbots, killer drones, hacks, glitches, exploitations, and manipulations. In this context, there is a very urgent need for the regulation of autonomous weapons [26], and a number of associations (e.g., the Future of Life Institute) are developing policy actions to monitor progress towards sustainable AI [27]. The 23 Asilomar AI principles [28] constitutea good start for a global shared AI vision of equitable benefits and prosperity for all without compromising people’s rights, privacy, autonomy, dignity, and freedom.

Thecurrent guidelines of the European Union (EU) [29] and UNESCO [30] on trustworthy AI can be adapted for other regions. Here, it is important to note that such guidelines should focus on concrete AI applications, rather generally on AI technology; this would both enable an adequate framework to enforce legal consequences and the proper environment for AI research and development. The guidelines and good practices of ethical AI in a human context [31] have been advocated with direct impacts on thelife and future of citizens. AI practitioners should consider the long-term implications and impacts for AI applications and data-driven innovations to be aligned with the SDGs, such as AI-enabled climate actions. AI applications should be ethics-driven, context-aware, and inclusive for use responsibly. Globally, the field needs intergovernmental regulatory harmonization and open policy for data transportability. We need shared visions and wisdom to keep AI beneficial to mankind [32] by ensuring that machines or robots understand, adopt, and retain humanity's goals. An additional SDG of Digital Technologies Serving People and the Planet (SDG18) has been advocated by the Montreal Statement on Sustainability in the Digital Age [12]. This is to ensure that the digital age supports people, the planet, prosperity, peace, and partnerships.

## Further implications

Robotics and AI have been applied in bionics to augment and enhance human capabilities. This is particularly meaningful for handicapped people to acquire artificial limbs and regain sensory perception. In this respect, human capability can be enhanced alongside the advancement of robotics. On the other hand, it remains challenging to create a robot with human-like dexterity and mobility for common tasks that we often take for granted, such as grabbing a drink from the fridge. This requires visual perception through computer vision in an unstructured environment in which the robot will take time to process and learn. This is because a robot can only “see” binary numbers, unlike human perception. The good news is that once computer intelligence is achieved, it can be easily replicated through cloud deployment to all machines or robots.

Although we are still far from sci-fi humanoids, many AI scientists are striving for a person-like robotic system with a body and mind analogous to those of a human being that can pass the Turing test. To achieve this, advancements have been made in chatbots, gaze tracking, object detection, emotion detection, and copying of the human sensorium. Humanoids are everimproving with advanced materials such as carbonfiber skeletons and Kevlar muscles equipped with pneumatic actuators using compressed air that is analogous to the human musculoskeletal system with proprioceptors. The goal is to create a virtual mind of a humanoid beyond knowledge, skills, and language towards an ultimate conscious machine that thinks on its own. Consciousness is related to awareness and a sense of being. This is how we fit in time and space with sensory memories that shape our manners. Synthsor synthetic humanoid robots can learn by imitating and generating artificial memories from texts via natural language processing (NLP), which can provide backstories for the creation of new memories in becoming a conscious machine [33]. Achieving a deep understanding ofthe neural code in human-brain science will have profound implications for the mind of a synth as revolutionary as deciphering the genetic code for biology.

## AI and humanity

In the process of advancing humanoids, AI experts appreciate more about humanity in terms of intuition, creativity, and empathy that are lacking in AI. Human-level common sense poses the greatest challenge in AI programming. In this regard, an AI transformation should be considered complementary instead of a total replacement of human society. Reaching the technological singularity [34] was once a fantasy. In the future, we might succeed in creating digital intelligent life on Earth while the SETI program is searching for extraterrestrial intelligence via the Allen Telescope Array (ATA). They use AI to teach machines to learn from mining vast volumes of data from the universe and looking for the exceptions to the norm. Synthetic superintelligence will transcend biological intelligence, resulting in unforeseeable changes to human civilization. Will this be a new dawn of human society and moral principles? Who will be responsible for robotic crimes? Will there be robot rights when they can be considered their own being with volitions? How about synth-ethics? By then, will we be pondering existential questions of humanity if we can upload our minds into the digital self?

Meanwhile, AI technology is already rapidly changing the world in terms of how we live and work. AI can create new opportunities and potentially fill the gaps of work that nobody wants to do. However, the labor market has been greatly impacted by reduced dependency on laborers, resulting in the unemployment of low-skill workers and widening the gap of society, which needs urgent attention in many countries [20].

## Conclusions

There is no doubt about the importance of AI. Its ramifying impacts depend on how the advancement of the field can be successfully translated into real-life applications with realistic and practical implementation without harming the planet and society. We must acknowledge that not all countries can afford to provide a social-safety net for the foreseeable income inequality between AI technology haves and have-nots. Harmonizing global guidelines and regulatory frameworks remains a great challenge that constraints worldwide AI application. These regulations should focus on specific applications of AI to facilitate technological development and legal enforcement. In this context, interpretability of AI [35] can really facilitate both the implementation and the audit of AI technology in a progressively wider range of areas. In a utopian world of a global welfare state, all AI applications should fall under the positive vision of shared prosperity to avoid destructive conflicts or wars between countries. Hopefully, AI-based technology can help to save the planet and ourselves for the betterment of humanity before the tipping point of global destruction.
